# Recovery time and heart rate variability following extreme endurance exercise in healthy women

**DOI:** 10.14814/phy2.13905

**Published:** 2018-10-31

**Authors:** Robert M. Gifford, Christopher J. Boos, Rebecca M. Reynolds, David R. Woods

**Affiliations:** ^1^ Centre for Cardiovascular Science Queen's Medical Research Institute Edinburgh Edinburgh; ^2^ Defence Medical Services Lichfield United Kingdom; ^3^ Department of Cardiology Poole Hospital NHS Foundation Trust Poole United Kingdom; ^4^ Centre for Postgraduate Medical Education Bournemouth University Bournemouth United Kingdom; ^5^ Research Institute, for Sport, Physical Activity and Leisure Leeds Beckett University Leeds United Kingdom; ^6^ Northumbria and Newcastle NHS Trusts Wansbeck General and Royal Victoria Infirmary Newcastle United Kingdom; ^7^ University of Newcastle Newcastle Upon Tyne United Kingdom

**Keywords:** Exercise, heart rate variability, women

## Abstract

The relationship between autonomic function and recovery following prolonged arduous exercise in women has not been examined. We undertook an exploratory study that aimed to examine the temporal change in linear and nonlinear measures of heart rate variability (HRV) following prolonged arduous exercise in the form of first all‐female (mean age 32.7 ± 3.1 years) team to attempt an unassisted Antarctic traverse. HRV analysis was performed before and 1, 4, and 15 days postexpedition. The traverse was completed in 61 days. There was a significant paired reduction in heart rate, LnLF, LF:HF, DFA *α*1 between baseline and 15 days postexercise in the same environment. Conversely, RMSSD, LnHF and HFnu, SD1:SD2, and SampEn significantly increased. DFA *α*2 levels significantly fell from baseline to Day 1 postexercise. In conclusion, we observed a significant latent increase in relative parasympathetic dominance and RR interval irregularity at 15 days post prolonged arduous exercise, versus pre‐exercise baseline, in a group of very fit and healthy adult women.

## Introduction

Autonomic control plays a key role in exercise performance and recovery and is highly dependent on exercise intensity, type, duration as well as background fitness (Levy et al. 1998; Kluess et al. 2000). The measurement of heart rate variability (HRV) can provide a unique and noninvasive insight into autonomic balance and relative sympathetic‐parasympathetic output in humans (Task Force 1996; Prinsloo et al. 2014; Kingsley and Figueroa 2016). The use of HRV in sport has emerged from being predominantly a research‐based tool to its current clinical role in the monitoring of cardiovascular fitness and the prevention of over training and postexercise fatigue (Le Meur et al. 2013; Plews et al. 2013; Kiviniemi et al. 2014; Prinsloo et al. 2014 Jiménez Morgan and Molina Mora 2017). Whilst the exercise‐HRV literature is dominated by traditional time and frequency domain measures, useful nonlinear and composite HRV parameters have emerged. The relationship of such parameters to recovery following exercise has not been widely studied (Sassi et al. 2015; Bellenger et al. 2016).

Published exercise‐HRV research has been largely focused on the effects of high intensity short duration (<1 h) exercise and the early postexercise recovery period (minutes to <12 h) (Hoshi et al. 2017). There has been far less emphasis on the effects of prolonged and arduous exercise and associated recovery on HRV, and little evidence is available from women. Autonomic balance and HRV are known to differ between men and women; women have lower sympathetic output than men, which may be cardioprotective, inferring a recovery advantage following heavy exercise (Koenig and Thayer 2016; Boos et al. 2017). Thus, examination of female autonomic recovery and their potential resilience following arduous exercise is desirable (Du et al. 2005; Dutra et al. 2013; Schäfer et al. 2015; Koenig and Thayer 2016).

In this exploratory study, we aimed to investigate the temporal changes in traditional and nonlinear HRV parameters following prolonged arduous exercise in a cohort of healthy women. We also examined the relationship between HRV scores to background anxiety, which is known to affect HRV (Beck et al. 1988; Boos et al. 2018). We hypothesized that arduous exercise would lead to postexercise fatigue and increased sympatho‐vagal balance.

## Methods

### Ethical approval

All subjects underwent written informed consent. Ethical approval was received from the Ministry of Defence Research Ethics Committee (827MoDREC/17). The study was conducted in accordance with the Declaration of Helsinki.

### Subjects and exercise protocol

The participants consisted of six healthy adult women from the British Army, who were attempting the first all‐female unassisted Antarctic ski traverse expedition (Exercise ICE MAIDEN). All participants were required to have an unremarkable selection medical assessment including normal blood pressure and 12 lead ECG. They commenced the 1700 km ski expedition in November 2017, with each individual hauling a sled weighing up to 80 kg, with two resupply points *en route* (https://www.mountain-equipment.co.uk/blog/ice-maidens-antarctica-expedition/). The air temperature varied from −2°C to −40°C with an altitude of <3000 m elevation (start and end ~700 m; maximum 2950).

### Examination, heart rate variability and anxiety scores

At the time of enrollment, the subject height (Seca stadiometer 213, Seca, Birmingham, UK); and weight (Seca scales 874) were measured. HRV was measured 38 days prior to the 1700 km ski expedition in Camberley, UK (33 m altitude) (visit 1), 24 h after completion of the expedition at Union Glacier, Antarctica (700 m altitude) (visit 2), 4 days after the expedition in Punta Arenas, Chile (altitude 34 m) (visit 3) and 15 days after the expedition in Camberley, UK (visit 4). Testing location ambient temperatures for each visit were 20°C (68°F), 16°C (61°F), 21°C (70°F), and 20°C (68°F), respectively. Humidity was between 80 and 88%. All HRV measurements were conducted fully rested in the early morning, postmicturition and prior to breakfast and caffeine. At visits 1 and 4, participants were positioned on an examination couch with the head and torso elevated 45°. At visits 2 and 3, participants were examined on fixed beds and positioned using pillows to a comfortable position of 30–45°. Participants were allowed to rest for 10–15 min before the reading was taken and told to relax and breathe normally during measurement. Lighting was controlled, and examination rooms were secluded from traffic or passersby. Visual and auditory stimuli were minimized prior to and during the examination. Talking was not permitted during ECG recording which was performed using a battery‐operated portable HRV devices (CheckMyHeart™, Daily Care Biomedical, Taiwan, http://www.dcbiomed.com) as previously described (Boos et al. 2017).

The CheckMyHeart™ device uses two surface electrodes to obtain a 5‐min single lead ECG capture (sampling rate of 250/sec) for HRV analysis. The electrodes were placed at the upper right sternal edge and cardiac apex, respectively. The stored ECG data were exported via USB hook up for initial offline data analysis (CheckMyHeart™ HRV analysis software V2.2, 2017‐12‐18 Daily Care Biomedical, Taiwan). All 5‐min ECG recordings were inspected to ensure the correct identification of all normal‐normal intervals (NNI). The NNI data were then imported into Kubios^®^ Premium HRV software (http://www.kubios.com; ver.3.0.2) for detailed analysis **(**Tarvainen et al. 2014).

Time and frequency domain measures of HRV were calculated according to the HRV Task Force Guidelines as previously (Task Force 1996). We recorded the average heart rate and the time domain RMSSD (root mean square of successive differences) and pNN50% (the number of NN intervals that differ by >50 msec divided by the total number of NN intervals). Spectral HRV parameters were calculated using the nondetrend IBIs by fast‐Fourier‐transformation (FFT). We reported the LF (low‐frequency) (0.04–0.15 Hz) and HF (high‐frequency) power (0.15–0.40 Hz) and the LF/HF ratio (Task Force 1996). Due to skewed distributions, LF and HF power were transformed by natural logarithms (ln). In order obtain greater insight into the relative HF power, it was reported as normalized units of HF (HFn.u.), which was calculated as HF/(LF + HF) (Task Force 1996; Boos et al. 2018). Poincaré plots were used to measure the standard deviation (SD) of short term (SD1) and long term (SD2) HRV and their ratios (SD1:SD2), which measure the unpredictability of the RR time series (Shaffer and Ginsberg 2017).

Nonlinear HRV assessment was examined as previously described (Sassi et al. 2015; Boos et al. 2018). Sample entropy (SampEn) and the short (*α*1, 4–12 beats) and long‐term detrend fluctuation analysis (DFA) slopes (*α*2, 13–64 beats) (Sassi et al. 2015) (Task Force 1996) were also measured. SampEn is a measure of the regularity and fluctuation of a time series with lower values representing less complexity and greater regularity (i.e. lower variability‐HRV) over successive RR intervals in a time series (Richman and Moorman 2000). DFA detects the simple correlations and self‐similarity between successive RRIs over nonstationary time scales with *α*1 reflecting the slope over shorter fluctuations and *α*2 over longer time periods (Shaffer and Ginsberg 2017). Lower correlation and hence DF *α*1 and *α*2 values tend to reflect higher HRV.

As anxiety is well known to influence HRV, all participants completed Beck Anxiety Inventory (BAI) self‐reporting questionnaires at baseline and 15 days postexercise in the UK. The BAI consisted of 21 questions, with each answer being scored from 0 (not at all) to 3 (severely). A score of ≤10 is normal and >10 indicates the presence of anxiety of increasing severity (Beck et al. 1988).

### Statistical analysis and sample size calculation

Data were analyzed using GraphPad InStat version 3.05. Results are presented as mean ± standard deviation (SD) for all continuous data. Owing to the small sample size comparison of continuous data across the four time points was performed using a nonparametric Friedman test. Similarly, paired differences in HRV between baseline pre‐ and post‐exercise were examined using Mann–Whitney nonparametric Tests. Correlations of continuous data were assessed using Spearman rank correlation coefficients (*r*) and 95% confidence interval. A two‐sided *P*‐value of <0.05 was considered as significant for all analyses.

Du et al. (2005) observed significant fall in heart rate and temporal changes in HF and LF power in HRV in the early recovery period (up to 30 min) following a maximal‐effort treadmill test among six elite adult female (aged 32–40) marathon runners. There were marked differences in all examined time and frequency domain parameters on comparisons of pre‐ and post‐exercise values. We anticipated that with a similar sample size of six females undergoing far greater intensity exercise and longer postexercise follow‐up, we would have sufficient power to discover any paired changes in HRV across the four measured time points (including pre‐ and post‐exercise).

## Results

### Subjects

The average age of the women was 32.7 ± 3.1 (range: 28–36) years with a mean body mass index of 24.9 (1.2). They were all Caucasian and nonsmokers. All participants completed the expedition together in a record time of 61 days. In their final 2 days, their exercise intensity was maintained having cross‐country skied 38.2 km (10 h day 60) and 29.4 km (8 h, day 61), respectively.

### Changes in heart rate variability

We observed significant changes in heart rate and HRV in the post arduous exercise recovery period versus pre‐exercise baseline (Table 1). Heart rate, LnLF, LF:HF, DFA*α*1 were significantly lower on paired comparisons between baseline and 15 days postexercise in the same environment. Conversely, RMSSD, LnHF and HFnu, SD1:SD2 and SampEn significantly increased. DFA*α*2 levels were significantly lower at Day 1 postexercise versus pre‐exercise baseline (visit 2). All the HRV measures significantly correlated with heart rate and each other except for DFA *α*2.

**Table 1:** Changes in heart rate and Heart rate Variability

	Pre‐exercise		Postexercise		*P*‐value
	Baseline (Time 1)	Day 1 (Time 2)	Day 4 (Time 3)	Day 15 (Time 4)	
Heart rate	65.9 ± 10.0	66.4 ± 10.0	60.6 ± 9.6[Link]	61.0 ± 9.7[Link]	0.03
pNN50,%	24.1 ± 19.2	35.2 ± 28.1	30.4 ± 32.0	42.1 ± 24.1	0.28
RMSSD	47.1 ± 20.9	59.7 ± 39.1	55.3 ± 40.7	62.9 ± 24.8[Link]	0.43
SD1:SD2	0.30 ± 0.06	0.57 ± 0.21	0.56 ± 0.21	0.68 ± 0.30[Link]	0.07
LnLF	7.33 ± 0.95	6.09 ± 0.82	6.17 ±1.78	6.18 ± 1.01^1^	0.13
LnHF	6.29 ± 1.34	6.62 ± 1.68	6.39 ± 1.72	7.10 ± 1.04[Link]	0.33
LF:HF	3.75 ± 2.80	1.88 ± 3.54	2.54 ± 4.77	0.41 ± 0.10[Link]	0.10
SampEn	1.31 ± 0.15	1.59 ± 0.60	1.61 ± 0.32	1.75 ± 0.29[Link]	0.04
DFA*α*1	1.24 ± 0.28	0.88 ± 0.50	0.82 ± 0.42	0.69 ± 0.16[Link]	0.09
DFA*α*2	0.98 ± 0.14	0.64 ± 0.12[Link]	0.87 ± 0.04	0.79 ± 0.28	0.01

pNNI%, Percentage of successive RRs >50 msec; RMSSD, root mean square of successive differences; SD, standard deviation; n.u., normalised units; LF, Low frequency; HF, high frequency; DFA, detrend fluctuation analysis; *P*‐value refers to the results of the Friedman test comparing all four time points for each parameter.

Refers to a significant paired change versus baseline.

### Anxiety scores

The average BAI scores were consistently within the normal range and a nonsignificant fell from baseline (8.3 ± 5.8) to Day 15 postexercise (5.5 ± 3.5; *P* = 0.63). There was no significant relationship between BAI scores and measures of HRV.

## Discussion

This is the first study to investigate the influence of prolonged recovery time (days) on HRV following extreme arduous exercise in women. We observed significantly greater HF power and RR interval irregularity and lower heart rate and LF:HF power at 15 days postexpedition compared with baseline. There were no significant changes in anxiety scores between baseline and postexercise.

Whilst regular exercise is well known to improve cardiovascular fitness it is physically demanding. These demands are heavily influenced by the individual and their background fitness level as well as the mode, intensity, and duration of exercise (Hautala et al. 2001;. Haddad et al. 2009; Silva et al. 2018). These factors are also of fundamental importance to the speed and efficacy of postexercise recovery. With prolonged and arduous exercise, the physical demands are obviously greater as is the associated recovery period and degree of postexercise fatigue (Hautala et al. 2001; Haddad et al. 2009). The identification of postexercise fatigue is important as it leads to reduced exercise performance, over reaching, and a greater risk of injury and over training. Recognised markers include sustained alterations in metabolic and biochemical markers, reduced (vs. pre‐exercise) physical and cardiac performance, and cognitive function (Kobayashi et al. 2005; Anderson et al. 2016; Silva et al. 2018). The duration of these changes is highly variable (hours to weeks) and depend on several factors including the duration and intensity exercise, the environment (temperature and humidity), the markers examined, and the population (e.g. age, sex, and background fitness) (Buchheit et al. 2007; Le Meur et al. 2013; Prinsloo et al. 2014; Bellenger et al. 2016; Ramos‐Campo et al. 2016; Boos et al. 2018).

The measurement of HRV is a noninvasive method used to examine training status, “exercise readiness” and postexercise fatigue. Whilst short‐term, postexercise HRV changes (<4 h) have been described, long‐term recovery responses have been less well studied, particularly following supramaximal, arduous exercise (Buchheit et al. 2007; Bellenger et al. 2016). The immediate postexercise HRV recovery pattern is typified by an immediate decrease in HRV markers of parasympathetic activation (e.g. RMSSD, SD1 and HF power) and heart rate, over minutes to hours. The postexercise reduction in sympathetic output and return to the pre‐exercise state is more gradual; it has been suggested that persistent sympathetic activation for up to 72 h signifies overtraining and fatigue (Haddad et al. 2009).

In this study, we examined traditional markers of parasympathetic activation (RMSSD, SD1 and HF) and relative sympathetic dominance (LF:HF power), in addition to several nonlinear HRV parameters including SampEn, DF*α*1, and DF*α*2. Several clear trends emerged. There was a significant trend to higher time domain measures of HRV (RMSSD, pNN50%, and SD1:SD2) and lower heart rate at 15 days postexercise versus pre‐exercise. This is likely to be indicative of improved fitness and cardiac adaption to physical training enhanced cardiac‐parasympathetic activity (higher RMSSD) and a reduction in sympathetic tone (LF:HF power) (Da Silva et al. 2014; De Freitas et al. 2015). The paired (baseline vs. 15 days postexercise) increase in SampEn, DFA*α*1, and *α*2 indicates that physical training led to reduced regularity (i.e. higher HRV) and greater complexity in the cardiac inter‐beat signal (Mourot et al. 2004; Shaffer and Ginsberg 2017; Boos et al. 2018). Whilst there was an upward trend in HRV from days 1–15, there was no evidence that the participants experienced postexercise fatigue.

The HRV profile in women is known to have a reduced sympathetic component of cardio‐autonomic modulation than is seen in men (Koenig and Thayer 2016). This could help explain why women have a lower risk of exercise related sudden cardiac death and could plausibly offer a recovery advantage over male counterparts (Dutra et al. 2013; Schäfer et al. 2015). Despite these differences, women have been under‐represented in exercise‐HRV research and hence a study exclusively of women following extreme exercise was a key premise of this study. In what was probably the first study to examine heart rate and autonomic recovery following arduous exercise, Hautala et al. (2001) examined HRV in nine healthy men (age 36.2 ± 10.6 years), before and after a 75 km cross‐country skiing race. R‐R intervals were recorded 24 h pre‐exercise and 48 h postexercise. There was no difference in heart rate or autonomic function at 24 h postexercise, however by 48 h postexercise, there was a significant decrease in heart rate, LF and the LF:HF power, and increased standard deviation of NNIs and HF power. More recently, Murrell et al. (2007) examined seven healthy adult men and women (average age 32 ± 10 years) 2 weeks before and at 1–4 h after and 48 h after a mountain marathon (42.2 km; cumulative gain 1000 m; ambient temperature 15°C (59°F); completion time, 261 ± 27 min). Compared with pre‐exercise, heart rate, LF and LF:HF power were notably higher in the early postexercise period (1–4 h), however there were no differences between pre‐exercise and 48 h postexercise. It is worth noting that both the method of breathing (controlled vs. spontaneous) and posture (whether supine, seated or upright) were significant confounders, underlining the importance of consistent HRV protocols. In another study, 25 men (36.7 ± 1.53 years) were investigated 1 day before, 1 h, and 1, 3 and 7 days after an Ironman ultra‐endurance event (3.9 km swim, 180.2 km cycle, 42.2 km run) (Gratze et al. 2005). The sympathetic dominance and higher heart rate observed 1 h postexercise had resolved within 3 days; heart rate and LF power decreased and HF power increased to pre‐event levels.

Our study adds to previous work by focussing exclusively on women, examining a longer postexercise recovery period (15 days) and the inclusion of nonlinear HRV measures. Whilst our early (1 and 4 days) postexercise findings are consistent with extant studies, the increased HRV 15 days postexercise is intriguing given that the participants had rested for 15 days following 61^ ^days of arduous exercise. These significant improvements 15 days postexercise compared with pre‐exercise HRV support both improved fitness and sustained recovery.

This study has several limitations. The sample size was very small, which is not surprising given the nature of expedition. Only three women had ever previously completed an Antarctic traverse and two of these were assisted by kites. This study was therefore exploratory. Our subjects were very fit and potentially elite athletes, hence the translation of these findings to less conditioned individuals is uncertain. The fact that we could study six women who all completed the expedition and measure four HRV time points over two continents is a significant accomplishment. Baseline HRV was only performed in the UK and several weeks before the study commencement for logistical reasons. It would have been insightful to have had baseline pre‐exercise HRV measures undertaken in Antarctica prior to exercise commencement. Similarly, whilst HRV was measured in the early morning prior to caffeine intake the environment in which the HRV was measured varied by being performed in the UK at baseline and study end (Day 15 postexercise) and in Antarctica on days 1 and 4 postexercise. While we attempted to replicate 45° semi‐recumbent positioning, the use of beds rather than examination couches in Antarctica and Chile may have affected the results. However, at both pre‐exercise and 15 days postexercise, an examination couch was used with subjects at semi‐recumbent at 45°, so that the key finding of improvement beyond pre‐exercise levels is unaffected by positioning.

In conclusion, in this first all‐female trans Antarctic ski crossing, prolonged arduous exercise was associated with a latent increase in relative parasympathetic output and RR interval irregularity (HRV).

## Conflict of Interest

None declared.
